# Biotribology of Synovial Cartilage: A New Method for Visualization of Lubricating Film and Simultaneous Measurement of the Friction Coefficient

**DOI:** 10.3390/ma13092075

**Published:** 2020-04-30

**Authors:** Pavel Čípek, Martin Vrbka, David Rebenda, David Nečas, Ivan Křupka

**Affiliations:** Faculty of Mechanical Engineering, Brno University of Technology, 616 69 Brno, Czech Republic; Martin.Vrbka@vut.cz (M.V.); David.Rebenda@vut.cz (D.R.); david.necas@vut.cz (D.N.); krupka@fme.vutbr.cz (I.K.)

**Keywords:** biotribology, cartilage, reciprocating tribometer, friction, lubrication, fluorescence microscopy

## Abstract

A healthy natural synovial joint is very important for painless active movement of the natural musculoskeletal system. The right functioning of natural synovial joints ensures well lubricated contact surfaces with a very low friction coefficient and wear of cartilage tissue. The present paper deals with a new method for visualization of lubricating film with simultaneous measurements of the friction coefficient. This can contribute to better understanding of lubricating film formation in a natural synovial joint. A newly developed device, a reciprocating tribometer, is used to allow for simultaneous measurement of friction forces with contact visualization by fluorescence microscopy. The software allowing for snaps processing and subsequent evaluation of fluorescence records is developed. The evaluation software and the follow-up evaluation procedure are also described. The experiments with cartilage samples and model synovial fluid are carried out, and the new software is applied to provide their evaluation. The primary results explaining a connection between lubrication and friction are presented. The results show a more significant impact of albumin proteins on the lubrication process, whereas its clusters create a more stable lubrication layer. A decreasing trend of protein cluster count, which corresponds to a decrease in the thickness of the lubrication film, is found in all experiments. The results highlight a deeper connection between the cartilage friction and the lubrication film formation, which allows for better understanding of the cartilage lubrication mechanism.

## 1. Introduction

Painless movement, i.e., the proper function of our joints, is very important in the active life of a human. Despite advanced medicine, there are many diseases of natural joints (osteoarthritis, arthrosis, arthritis, etc.) that lead to degradation and soreness of these joints [1,2]. The extent of the disease depends on many factors and may result in incurable, irreversible damage of natural joints. In this case, a natural joint has to be replaced by an artificial one. However, these replacements have a limited lifetime, which is a problem especially for young active people [3]. When the artificial joint is worn, it has to be replaced by a new one. This process cannot be repeated many times because of the adverse impacts on human health (bone degradation due to a thorn replacement, mental intensity of the operation for the patient, etc.) [4,5,6].

Some of the methods of the treatment of damaged natural joints are non-invasive methods, e.g., viscosupplementation (the supplement (a gel-like fluid called hyaluronic acid) is injected into the joint gap) [7]. The general effort of non-invasive treatments is to postpone the operation of total endoprosthesis as long as possible. Viscosupplementation is a treatment method allowing to restart the lubrication process in a damaged synovial joint, which temporarily stops or at least stabilizes the degradation of natural cartilage [7,8,9]. So far, the function of the supplements has not been completely understood, and their effects are not fully guaranteed for individual patients [10,11,12,13]. To better understand these issues, it is necessary to describe the cartilage lubrication. The cartilage and synovial fluid properties are the basis of articular joint lubrication [14,15].

The cartilage together with a natural lubricant (synovial fluid) are very important parts of the synovial joint for tribology investigation. The cartilage surface along with the synovial fluid distribute load between the opposite bones [16]. Due to the very low elastic modulus of cartilage tissue and unique synovial fluid properties, the contact pressure is distributed over a large area of the cartilage surface; therefore, the friction coefficient (CoF) is very low [17]. The cartilage tissue is formed largely by water, type II collagen fibers, hyaluronic acid (HA), lubricin, etc. [16,18,19,20]. It is a sectionporous fabric with a varying structure through the thickness of the cartilage divided into three zones [16]. The structure varies especially in the composition, shape, and orientation of fibers [21,22,23,24]. Due to a low cell density, the cartilage tissue is nourished through the synovial fluid. The synovial fluid of natural joints normally functions as a natural biological lubricant, as well as a distributor of nutrients to the cartilage tissue [25]. The synovial fluid mainly consists of HA, phospholipids, proteoglycans, etc. [26,27,28,29]. The structure of cartilage contains negatively charged particles, which ensure sucking of water (synovial fluid) to the structure pores [30]. The accumulation of water in the structure of cartilage is the principle of the unique lubrication system, and many explanatory theories are based on it. In general, these properties are the basis of the lubrication processes in the synovial joint.

The research exploring the tribology of natural joints is focused on various techniques and methods. Some studies are concerned with artificial joints (joint replacement) and observation of their lubrication [31,32]; others deal with biochemical analysis of materials for the manufacturing of joint replacements and their impact on the lubricating processes [33]. The study experimentally tests the chitosan coating of the artificial prothesis, and the results show that this method causes a decrease in CoF. This is caused by the adsorbed protein layer formed on the surface because the chitosan coating binds the proteins. The publications [34,35] were focused on friction testing of artificial materials: hydrogels. Publication [35] tested the artificial cartilage from hydrogel, and publication [34] carried out friction tests with a lens from the hydrogel. The similar values of CoF as in natural cartilage were reported in both studies; however, the work in [34] showed that the friction forces were composed of three components (viscoelastic dissipation, interfacial shear, and viscous shearing). Other studies also dealt with the articular cartilage; however, their investigation was not directly focused on tribology. The work [36] carried out an extensive study focused on the treatment of mature cartilage, and the growth factor-induced therapy was evaluated using gradually carried out analyses. 

There are two main types of works describing the cartilage tribological performance. The first group of works deals with cartilage lubrication, while the second one is focused on the friction between the cartilage tissues. 

The first group is concerned with several mechanisms for the lubrication of articular cartilage including hydrodynamic lubrication [37], boundary lubrication [38,39,40,41], weeping lubrication [42,43], and boosted lubrication [40]. Other works of this group, which presented complementary information for already published results or describing new lubrication mechanisms, appeared in later years: hydration lubrication [44,45] and adaptive multimode [46,47,48]. These studies were focused on visualization of natural cartilage or hydrogels by fluorescent microscopy; their aim was to support and verify the above lubrication theories. A great influence of ϒ-globulin in the lubrication of hydrogel has been shown, but it depends on its concentration and the concentration of other lubricant components [35]. HA also seems to be very beneficial for the lubrication of cartilage [49]. It creates a gel-like layer on the cartilage surface and binds with chondrocytes contained in the cartilage structure. The penetration of the cartilage surface depends on the size of the particles; small particles penetrate the cartilage, while larger ones adhere on the cartilage surface and create a gel-like layer [50]. The fluid leakage from the cartilage structure has also been proven, which supports the presented lubrication theories. 

The second group of works is focused on cartilage friction. The number of “friction” studies is higher because the friction measurement methodology is better developed, and these works indicate well the understanding of the cartilage friction behavior. The behavior of cartilage lubricated by synovial fluid was presented in [51,52,53,54,55] showing that the synovial fluid reports a very low CoF. HA helps to lower CoF [54]; CoF grows with time [51,52,54,55,56,57,58]; CoF decreases with a rising load [51,57,58]; CoF is influenced by the type of movement depending on the cartilage sampling [52,59]; and the cartilage rehydration has a positive impact on CoF [60]. 

Obviously, there are many friction studies and also studies focused on the visualization of cartilage contact with a well indicated examined area, but there are no works allowing for simultaneous measurement of friction and visualization of cartilage contact. Fluorescence microscopy is a suitable experimental method for visualization of the cartilage contact owing to a very compliant material (cartilage) and the non-reflective surface of cartilage, which is not possible by traditional methods, e.g., optical interferometry. Another limitation of cartilage contact visualization is the non-conductivity of samples (cartilage-glass); therefore, the electrical methods cannot be used. Fluorescent microscopy is the only applicable method that allows for visualization of non-reflective, non-conductive, and compliant materials. The studies focused on the visualization of cartilage lubrication film are very limited, and the relationship with friction measurements is missing. Moreover, there is no work describing the connection between the friction in synovial joints and the visualization of cartilage contact in order to provide a better description of lubrication processes in the synovial joint. The present study explains the missing relationship to allow for a better understanding of lubrication in the synovial joint. The aim of this study is to carry out the visualization of cartilage contact by fluorescence microscopy simultaneously with the friction measurement and to explain the new methodology for the description of lubrication film in the model of the synovial joint. A similar approach to the processing of results has never been published. In this connection, a designed reciprocating tribometer and evaluating software are presented. The research study submitted offers a description of lubricating film formation in the model of the synovial joint, which can help to develop new treatment supplements along with understanding of their function.

## 2. Materials and Methods

### 2.1. Experimental Device

**Figure 1:**
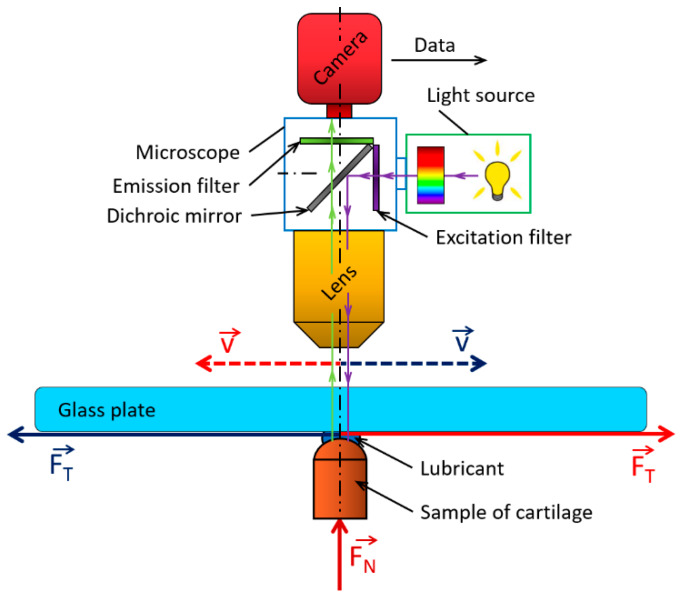
Schema of the experimental apparatus.

A unique design of the reciprocating tribometer was used; the scheme of the device is shown in Figure 1. This new design allowed for the in situ contact observation simultaneously with friction measurement. The design created was inspired by the concept in [60], where a similar design was used. A detailed description was published in the previous studies [61,62]. The use of the reciprocating tribometer simulates the compliant contact between the cartilage sample and the glass plate to create a simplified model of the synovial joint. The cartilage sample was situated under the glass plate in the static position. This arrangement allowed for the contact area visualization by the fluorescence optical system combined with a high-speed camera. The contact area was flooded with lubricant, and the lubricating bath was heated to the temperature of the human body. The glass plate was a moveable part with a reciprocating motion. Load was applied through the cartilage sample, and the lever measured friction forces. The operating conditions (stroke, sliding velocity, and load) could be modified. The tribometer was situated under the optical microscope based on fluorescence microscopy (Nikon, Eclipse NI, Minato, Tokyo, Japan).

**Figure 2:**
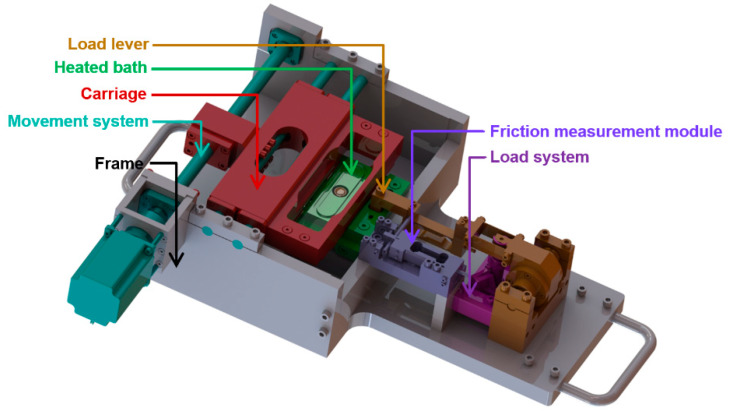
Digital model of the reciprocating tribometer.

**Figure 3:**
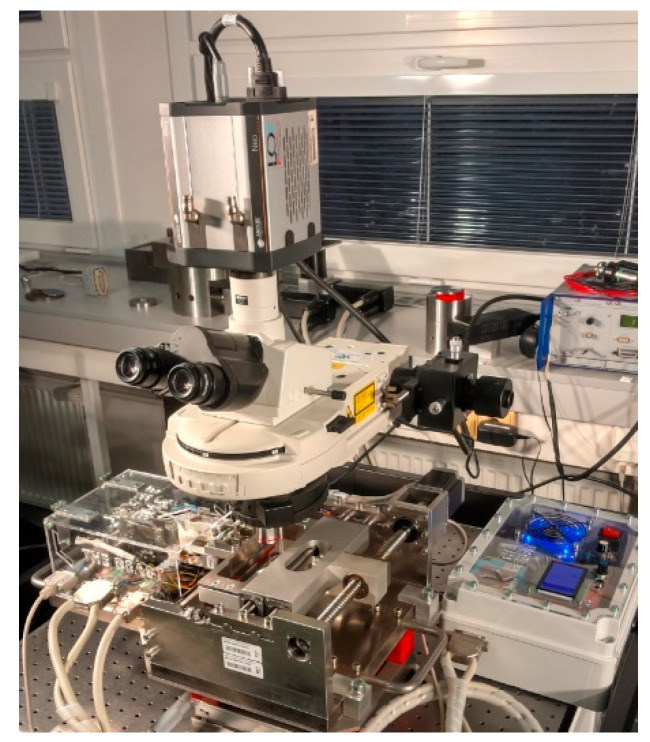
Experimental apparatus.

The experimental device is shown in Figure 2 and Figure 3. The basis of this concept was a rigid frame where the other components were mounted. The glass plate was fixed to the carriage, which was actuated using a combination of a ball screw and a stepper motor. A clearance-free and accurate reciprocating motion of the carriage was ensured by guide bars and the ball sleeve. The sealing between the glass plate and the heated bath prevented the leakage of lubricant from the bath. The sample was mounted at the end of the lever through which the load was applied and distributed by a linear stepper actuator. This actuator was placed under the lever, at the opposite end to where the sample was mounted. The load sensor was a part of the lever, and the friction sensor was connected in parallel to the lever. The c control system was based on Arduino, which controlled the movement and the loading system. The input parameters were entered through an LCD interface. The measuring system worked separately and was fitted with two single-point tensometric sensors to allow for recording of loading and friction forces. Force curves were saved to the data files; CoF was calculated therefrom.

### 2.2. Experimental Method: Fluorescence Microscopy

In this paper, the optical method based on the principle of mercury lamp inducted fluorescence was used. Fluorescence is the light emission of a substance; it is excited by light or other electromagnetic radiation. The fluorescence phenomenon can be described in three steps: excitation (the excitation photon emitted by the excitation light source is absorbed by the fluorophore contained in the fluorescent dye), excitation state period (dissipation of energy to ensure the emission of fluorescence), and emission (due to the dissipation of energy in the excitation state period, the photon emitted by the dye has a lower energy; therefore, it emits radiation at longer wavelengths). A full description of the fluorescence method was given in [63]. The mercury lamp emitting white light was used as an excitation source. The carousel with various excitation and emission filters were placed in front of the light source. For the purpose of this study, Fluorescein Isothiocyanate (FITC) and Tetramethylrhodamine (TRITC) filters were used; they allowed for a change in the wavelength of excitation and emission light, FITC (excitation on 490 nm, emission on 525 nm) and TRITC (excitation on 557 nm, emission on 576 nm). The double magnification lens was used for the observation of contact. The analysis of the apparatus was carried out, and the axial resolution was approximately 290 μm for the FITC filter and 320 μm for the TRITC filter. The field depth of the apparatus was approximately 200 μm, which was sufficient with respect to the sliding speed and estimated film thickness. Focusing of the image (contact) was carried out by the field diaphragm and slight re-focusing by the z-feed of the microscope. The focusing procedure was realized before each experiment, and it was immutable during the experiments. The fluorescence method was introduced and described in previous studies visualizing joint replacements for different material combinations [31,32]. The glass plate, through which the contact was visualized, was made from optical glass B270; therefore, the light excitation and emission were not affected. The scheme of the apparatus is shown in Figure 1. 

### 2.3. Specimens and Lubricants

**Figure 4:**
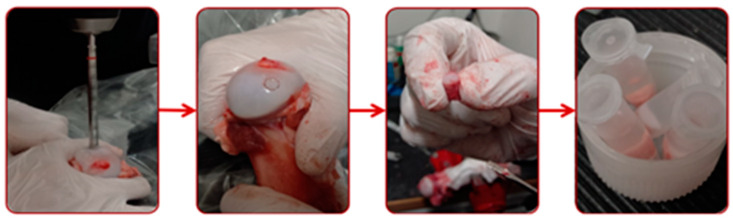
Sampling process.

Samples were removed from the femoral hip head of mature pigs. The sampling process was realized as early as possible after the slaughter of the animal. The samples were removed from the canopy of the most loaded site of the femoral head, which ensured the best mechanical properties. This site was selected for all sample bones to achieve a low deviation of mechanical and tribological properties. The samples were removed by a hollow punch with an internal diameter of 9.7 mm and stored in phosphate-buffered saline (PBS), deeply frozen (−20 °C), and defrosted immediately before the experiments. The same sampling procedure was used in [55,58] and verified in [64,65]. The sampling process is shown in Figure 4. The opposite sample to the cartilage was the glass plate, which fulfilled the immediate requirement for insight into the contact area. The dimension of the glass sample was 154 mm long, 43 mm wide, and 4 mm thick, which allowed for a sufficient stroke without leakage of testing fluid.

As an experimental lubricant, a model synovial fluid was used. The composition of the model synovial fluid corresponded to the physiological synovial fluid; see Table 1. Bovine serum (BS) albumin (powder, ≥96%; A2153, Sigma-Aldrich, St. Louis, MO, USA) was labelled by Rhodamine-B-isothiocyanate (283924, Sigma-Aldrich, St. Louis, MO, USA) in this case, and the other components were mixed without dye. The protein solution was further comprised of ϒ-globulin from bovine blood (powder, ≥99%; G5009, Sigma-Aldrich, St. Louis, MO, USA) and HA with a molecular weight of 1000 kDa. All these components were mixed in PBS solution. The lubricant degraded in air; therefore, the lubricant specimens were stored in a frozen state at −20 °C and defrosted immediately before the experiments. A single lubricant was used for each experiment, and after that, the lubricant sample was discarded. The duration of each experiment (see Table 2) was too short to degrade the lubricant sample. The other conditions of the experiments (especially contact pressure and temperature) did not speed up the degradation. 

### 2.4. Methodology and Conditions

**Figure 5:**
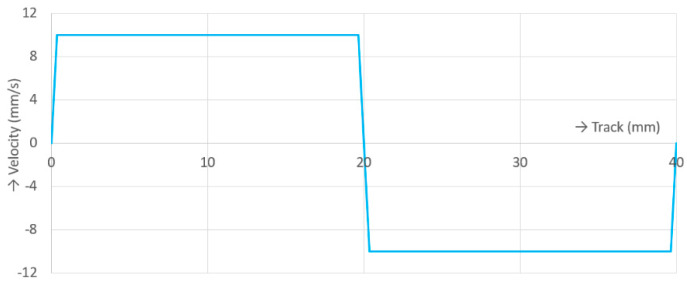
Sliding velocity trend.

A strictly-defined procedure of the preparation of each experiment was defined for adherence to the repeatability of the results as described in the previous study [61]. All experiments were carried out under the same experimental conditions; see Table 2. These conditions were defined based on the previous studies and inspired by natural synovial joints [20,66,67]. The contact pressure was approximately 0.8 MPa. The value of the contact pressure was determined by the Hertz theory. This value was strongly dependent on the modulus of elasticity of removed cartilage, which varied through all specimens. The contact pressure was applied as 10 N of load. Similar conditions were used for example in [54,60]. The tribometer allowed for a constant majority of the velocity trend, as shown in Figure 5, where one cycle of reciprocating motion can be seen. The constant part of the velocity trend was 96.5% of every cycle. 

**Figure 6:**
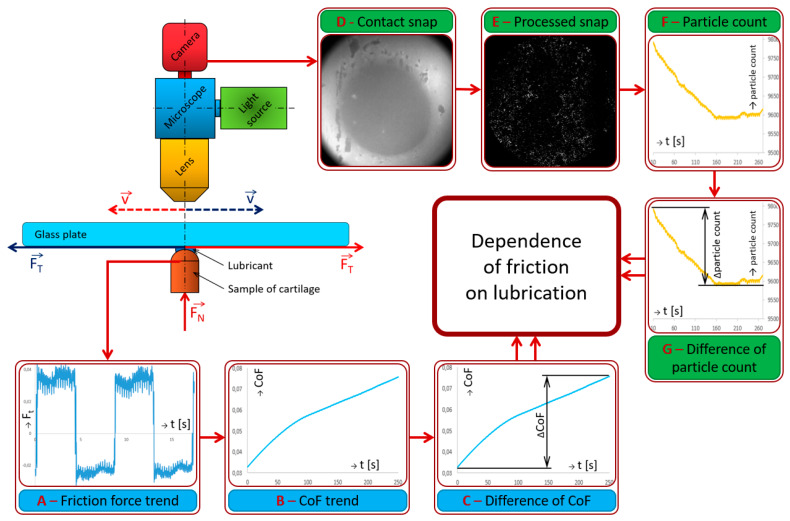
Evaluation schema. (**A**) Friction force trend (**B**) CoF trend (**C**) Difference of CoF (**D**): Contact snap (**E**) Processed snap (**F**) Particle count (**G**) Difference of particle count.

The output of each experiment was a force record and recorded snaps of the contact area. Each experiment had to be post-processed in two ways: the effect of the forces and the visualization (snaps of contact); however, both processed results were linked at the end of post-processing. The schema of the experiments’ evaluation is shown in Figure 6. The raw data from the effects of forces needed to be edited and filtered for further processing. Due to the preloaded friction sensor, the raw signal had to be offset in the first step (Figure 6A), and after that, CoF could be calculated (Figure 6B) (ratio of friction force and normal force). The complete procedure for evaluation of the effect of forces was published in [52]. The CoF trend was determined from each measurement, and the percentage difference of the CoF value from the beginning to the end was evaluated for each experiment; see Figure 6C. The percentage difference was determined from the average of the last 1000 values of CoF and subtracted from the average of the first 1000 values; this was the first evaluation parameter for the final evaluation. 

The record of contact from the camera was the second parallel way of input from the experiments. The raw record from the camera was exported to the particular snaps in the first step (Figure 6D). The snaps were in a resolution of 2560 × 2140 pixels, and the pixel size while using the double magnification lens was 3.75 micrometers. The individual snaps are processed by specially designed software, which filtered out the background of each snap and highlighted the lighter points; see Figure 6E. A description of the software is given in the paragraph below. The lighter points show the labelled proteins in the lubricant; this was a significant evaluation parameter. The background noise was caused by the lubricant, which was sucked in the cartilage pores. The particle count in the contact was an output of each snap. The dependence of particle count on time was an output of the entire record; see Figure 6F. The software processed every snap in every recording according to the input parameters. The difference between the initial and final particle counts in the contact was the second evaluation parameter for final evaluation; see Figure 6G. The dependence between the particles’ difference and the friction difference led to a lubrication description, especially the determination of the influence of synovial fluid individual components on the lubrication of cartilage.

**Figure 7:**
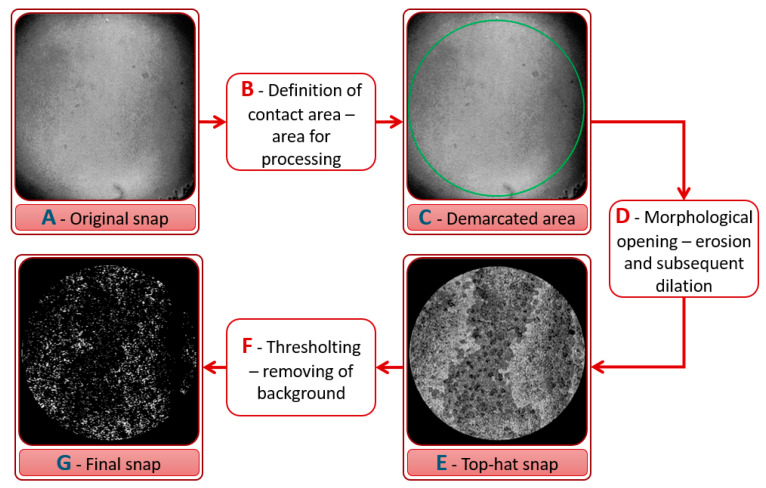
Snap processing diagram. (**A**) Original Snap (**B**) Definition of contact area-area for processing (**C**) Demarcated area (**D**) Morphological opening-erosion and subsequent dilation (**E**) Top-hat snap (**F**) Thresholting-removing of background (**G**) Final snap.

**Figure 8:**
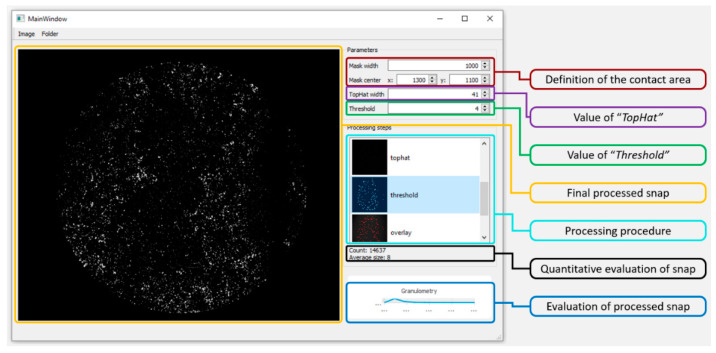
Evaluation software.

The processing of snaps was based on the unique software design (Figure 8) using the segmentation principle of image processing. Segmentation is the process of transforming the individual parts of the snap into meaningful regions or objects [68,69]. This software removed the background and highlighted the labelled proteins from snaps in several steps. The whole procedure of snap processing is shown in the diagram in Figure 7. First, the contact area was defined by a circle, Steps 7B and 7C, and the surrounding area was suppressed, i.e., the surrounding area did not enter the future processing. The contact area was defined based on visual observation; the contact area was clearly visible in each snap. In the second step, dual operations, erosion and dilation, were carried out (this combination is called morphological opening, Step 7D); therefore, at first, structuring element (SE) was searched in the examined area, and if SE was detected, the pixel was added into the center of SE (i.e., the examined area was reduced by the SE radius). After that, the overlap of SE in the examined area was determined. If SE, at least partially, overlapped the examined area, the center of the resulting area was added (i.e., the examined area was magnified by the SE radius) [69]. The opened snap was subtracted from the original snap (Steps 7D–E). Finally, thresholding was carried out; see Steps 7F and 7G in Figure 7; according to the threshold value, all points that were below the threshold were suppressed. This principle was used in [70]. The author used the morphological opening for processing of microscope snaps of a metallic alloy to highlight some parts of the snap and remove the background. 

A snapshot of the software with its description is shown in Figure 8. In the top right corner, there are fields for input parameters. Functions “Mask width” and “Mask center” define the circular area by which the size and position of the contact are defined. The “TopHat width” value defines sensitivity to the local snap maximum, and consequently, the areas for morphological opening are defined. The last box “Threshold” defines the threshold to determine the background. The processing steps of snaps are shown under the boxes with input parameters. The software determined the count of detected proteins and their average size; this is shown under the images with processing steps. The graph in the lower right corner describes the count and the size of particles found. The final processed snap is shown on the left side of the software window.

## 3. Results and Discussion

### 3.1. Verification and Calibration of the Method

**Figure 9:**
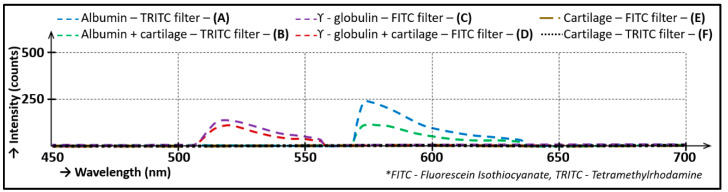
Lubricant emission.

The cartilage contact was verified by a spectrometer. The individual lubricants and their combinations with the cartilage were tested by spectral analysis. They were radiated using TRITC and FITC filters, and their emissions were monitored. The results are shown in Figure 9. Obviously, no emission was detected if the cartilage alone was excited using both filters, as shown by Curves E and F in Figure 9. The cartilage in combination with labelled lubricants always emitted only at wavelengths of the filter used, which was obvious from Curves B and D. The labelled lubricant alone also emitted only at wavelengths of the filter used (see Curves A and C); however, the emission was stronger than in the case of lubricant with cartilage. The chart in Figure 9 shows that the individual lubricants and cartilage combinations were not mutually affected throughout measurements because their emissions were offset relative to each other.

**Figure 10:**
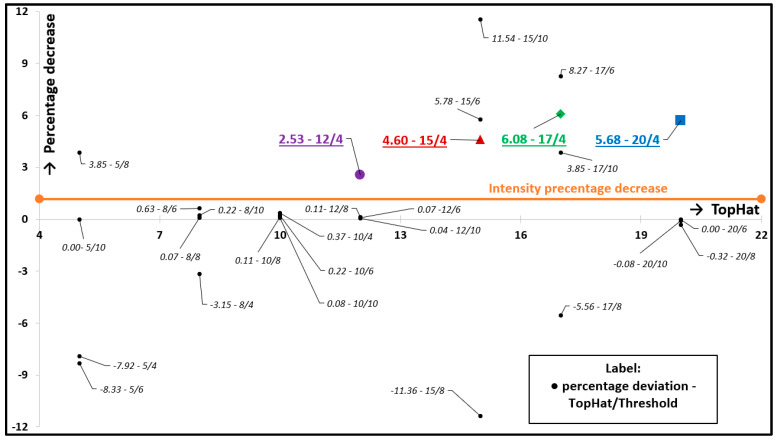
Knockout chart.

The software was calibrated based on the fluorescence intensity trend, which was directly exported from camera records. The recording device was a high-speed camera allowing for recording with a high framerate. The originally delivered camera software was used to export the intensity and to convert the video record into individual snaps. These snaps must then be processed by the newly designed software. A wide spectrum of combinations of input parameters for processing by this software was compiled; however, only a small section of the spectrum was selected using the knockout chart in Figure 10. This chart shows the entire spectrum of different combinations of software input settings. Each individual point represents one input combination, and the whole chart shows a percentage decrease of particle counts throughout the experiments. The total percentage decrease was determined similarly to the CoF difference (Section 2.4), specifically from the average of the last six cycles of particle counts, and it was subtracted from the average of the first six cycles. The value from the middle of each cycle was used for average calculation. The points, which report the opposite trends to fluorescent microscopy, were eliminated (points in Figure 10 under the red line). From the remaining trends, only the points reporting similar trends as the fluorescent intensity trend were used for the following step. 

**Figure 11:**
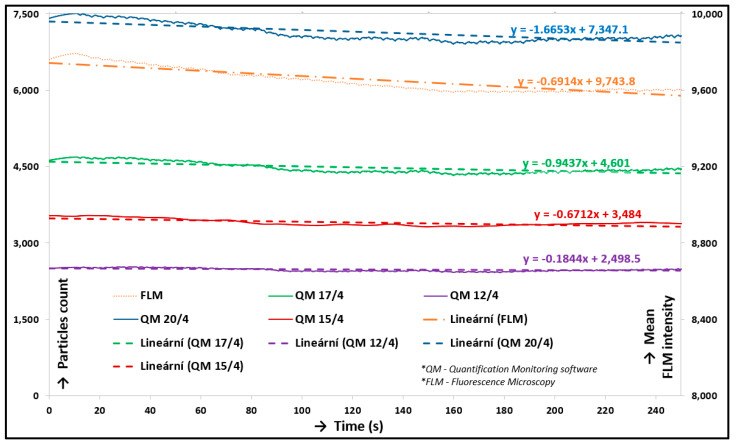
Comparison chart of individual input parameters.

In this step, the similarity of the percentage decrease of particle count trends and fluorescence intensity was compared. For the next step, only the results closest to the fluorescence percentage decrease trend were used; therefore, the two y axis graph was compiled. The y axis on the right represents fluorescence intensity (Fluorescence microscopy (FLM) in Figure 11). The y axis on the left represents the particle count. The x axis is time. This dependency is shown in Figure 11. These trends were interpolated by a linear curve, and the tangent directions of these curves were compared. The most similar settings were Quantification Monitoring software (QM) 15/4 and QM 17/4; both of them well described the intensity trend, and their tangent directions were the most similar ones. As the final setting of software, setting 15/4 was defined, which meant TopHat 15 and Threshold 4. However, this setting was valid only for measurement with labelled albumin. A new calibration was necessary for each measurement to validate the data from the new software. The independent calibrations of evaluation software were carried out for both model synovial fluids, and the determined calibration inputs are shown in Table 3. 

### 3.2. Friction and Lubrication in Cartilage Contact

**Figure 12:**
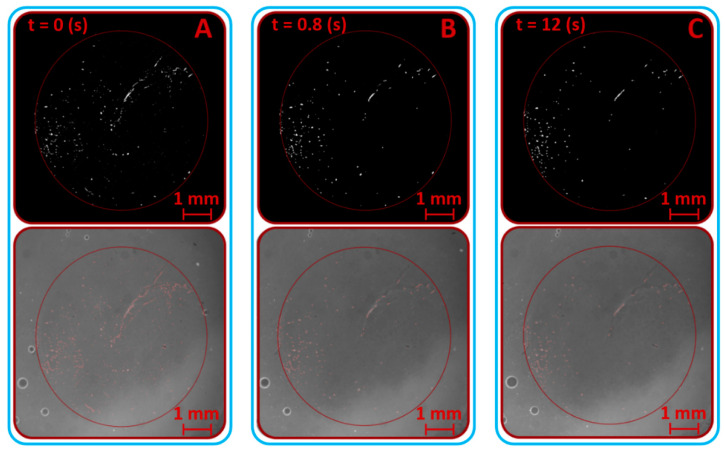
Loading of contact snaps – highlighted protein clusters by the software. (**A**)—protein clusters at the beginning of the experiment (in the time 0 s), (**B**)—protein clusters in the time 0.8 s, (**C**)—protein clusters at the end of experiment (in the time 12 s).

**Figure 13:**
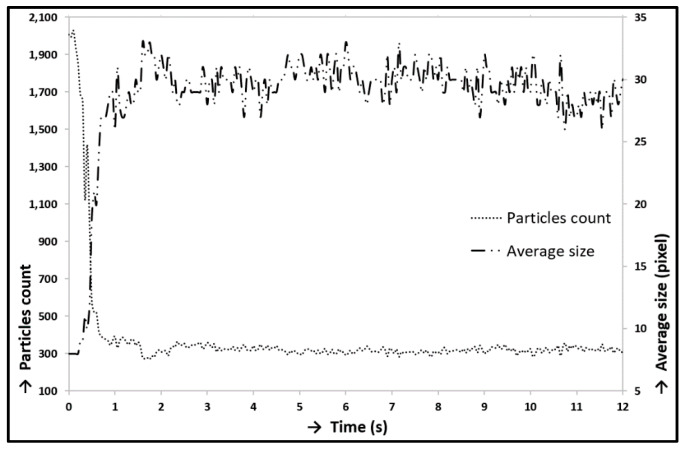
Loading of contact–particles count/average size.

**Figure 14:**
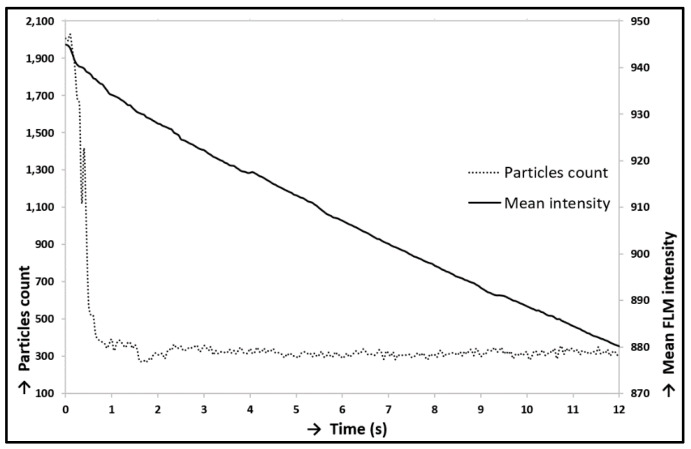
Loading of contact–particles count/FLM (fluorescence microscopy) intensity.

At first, prior to all experiments focused on lubrication analysis, the static experiment without reciprocating motion was carried out. The cartilage specimen was gradually loaded from the unloaded state to the load of 10 N by a linearly rising loading process. The contact was recorded simultaneously. This experiment was carried out only with the solution of albumin and PBS (Calibration Fluid 1; see Table 1). Figure 12 shows the states immediately before the contact was loaded (12A), immediately after the contact was loaded (12B), and after the contact was fully loaded (12C). The red circle in the snaps represents a cartilage contact area, and the white points represent clusters of labelled proteins; in this case, albumin. The lower snaps in Figure 12 show the original snaps, and the clusters of proteins are obstructed by thin red curves. The decrease of the count of particles in contact was obvious throughout loading. The particle count curve (Figure 13) showed a strong fall during the loading process; nevertheless, the following particle count trend did not vary much and reported only a slight decrease. A decreasing trend of particle count corresponded to a decreasing trend of fluorescence intensity (Figure 14). However, the size of protein clusters sharply rose during the loading process, i.e., the size of protein clusters increased with rising load. This trend corresponded to the contact snaps in Figure 12. Conclusions from these experiments confirmed the presence of protein clusters in cartilage contact and their squeezing out from the cartilage pore structure, and subsequently from the contact. This confirmed a decreasing trend of particle count and fluorescence intensity. A similar theory, the escape of proteins from the contact and synovial fluid from the cartilage tissue during loading, was suggested and described in [40,41,42,43]; nevertheless, this phenomenon was described only at the theoretical level in connection with lubrication theories. The study [71] described similar results, the extrusion of lubricant during load, but based on different experiments.

**Figure 15:**
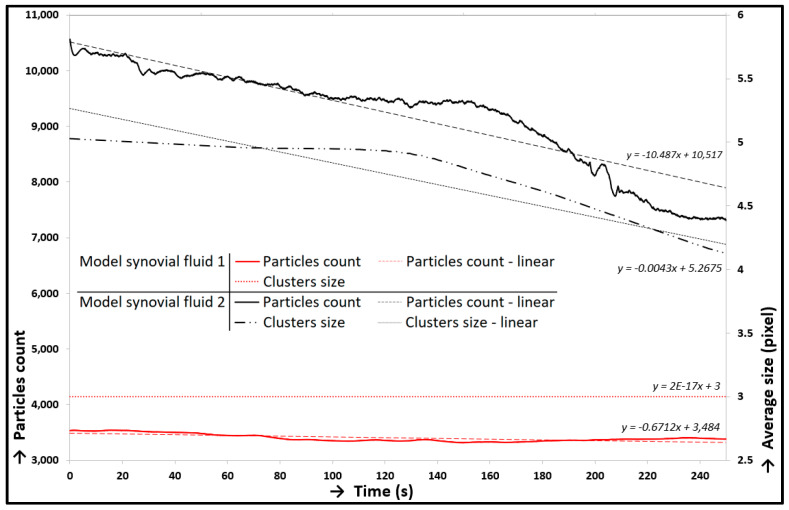
Particle count/average size/time-dependence: Model Synovial Fluids 1 and 2.

**Figure 16:**
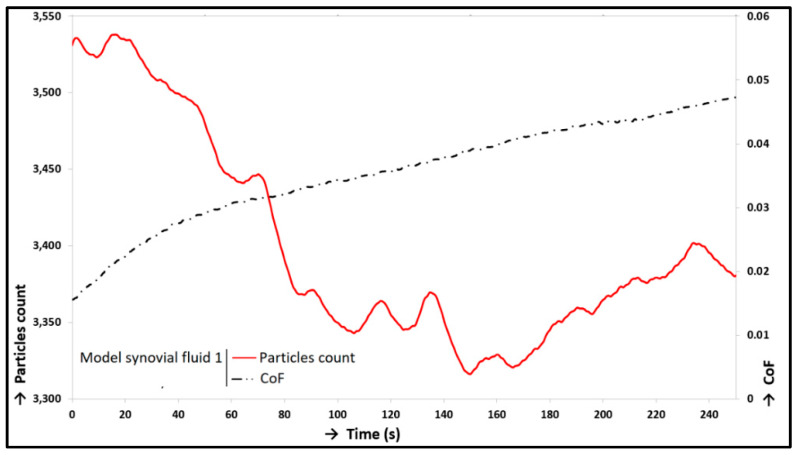
CoF trend: Model Synovial Fluid 1.

Two different measurements were carried out. The variation of experiments lied on two model synovial fluid variants with the same composition; nevertheless, a different labelled component was used (see Table 1). Both measurements were carried out under the conditions in Table 2 and focused on simultaneous recording of friction forces and visualization. The particle count trends and the trends of average size of protein clusters were evaluated and shown in Figure 15. Decreasing trends of particle counts were obvious from both curve trends. The percentage deviation of the decreases is given in Table 4. Model Synovial Fluid 1 showed a more significant decrease of particle count than Fluid 2 (see Figure 15) and also on deviation (Table 4). The average size of ϒ-globulin clusters (Model Synovial Fluid 2) decreased simultaneously with the decrease of the ϒ-globulin particle count; however, the average size of albumin protein clusters (Model Synovial Fluid 1) was unchangeable. The opposite deviations were shown by the simultaneous friction measurements. The deviation (percentage increase) of CoF trends was evaluated (see Table 4). This parameter was the second main output from the experiments. The correlation between CoF and particle count trends, measurement with Model Synovial Fluid 1, is shown in Figure 16. These trends implied the dependency between the initial increase in CoF and the decrease in particle count; i.e., the trends did not change much, and their changes were gradual. In the second measurement, a similar dependency was not distinct (measurement with Synovial Fluid 2, labelled ϒ-globulin). 

A comparison of particle count trends showed that the ϒ-globulin protein clusters were wiped off faster from the contact than the albumin; thus, the lubrication film was formed especially by albumin clusters. ϒ-globulin helped with the forming of the lubrication film at the beginning of the experiments, and the size of its clusters was much larger than that of the clusters formed by albumin; nevertheless, the lubrication film formed by albumin was more stable. The results suggested a greater influence of albumin proteins on lubrication because of their higher stability in contact, but the clusters formed by ϒ-globulin were more numerous. Similar conclusions were also presented in [35], but different lubrication compositions of the fluids were used with the same labelled components. The study suggested more ϒ-globulin clusters in the contact, but the trends of particle counts with both labelled components were different. This could be caused by the different composition of the experimental fluids, experimental conditions, and in particular different specimens (PVA hydrogel was used instead of cartilage samples). The decreasing trends of particle counts were shown in both measurements, although the trend of CoF was increasing. The measurement with Model Synovial Fluid 2 showed a steeper decline of particle counts, which indicated a connection with the growing trend of CoF (see Figure 15), but with a smaller impact on lubrication film forming. However, measurement with Fluid 1 showed a very gradual decrease of particle counts, which indicated a greater impact on lubrication, but a smaller impact on the increasing trend of CoF. These results showed that, in this case, the albumin proteins represented the main component responsible for lubrication film formation. In general, a decrease in particle counts in both measurements indicated squeezing out of lubricant from the contact area, i.e., weeping and squeezing out of protein clusters from the contact, which weakened the adsorbed film formed by the proteins. In general, the connection between the rising CoF trend and the decreasing trend of particle count pointed out that the adsorbed lubricating film on the cartilage surface, which was created by the protein clusters, was a key factor for the low friction in the contact and protected the cartilage surface from damage [50]. When the adsorbed lubricating film covered the cartilage surface, the motion (friction) took place inside or between the protein layers, i.e., lower CoF. The results showed that the albumin clusters were the basis of the lubricating film; they were adsorbed at first on the cartilage surface, and the ϒ-globulin clusters were bonded to them. The protein clusters were gradually wiped off from the contact; at first, the ϒ-globulin clusters and after that, the albumin clusters. This caused, in the extreme case, the contact of raw cartilage tissue, which damaged the cartilage tissue and increases the CoF. The presence of protein clusters was partly due to weeping of lubricant from cartilage pores, which was proven by the static loading experiment in correspondence with the lubrication theories published in [42,43]. These theories describe the cartilage lubrication based on the weeping of lubricant from cartilage pores during motion. The pores serve as a lubrication stack, and this is exhausted during motion, which causes a CoF increasing trend. In general, the decreasing trends of particle counts caused a decreasing thickness of lubrication film until the proteins were completely removed from the contact. According to the indications, it was expected that the carried-out experiment worked at the transition of the boundary and mixed lubrication regime depending on the value of CoF. A decrease in lubrication film led to an increasing CoF trend. Albumin had a greater effect on the CoF change from this perspective, but ϒ-globulin showed a greater change of particle count. Albumin showed a greater impact on lubrication in both cases (lubrication and friction). The values of CoF started approximately between 0.01 and 0.02 and ended at approximately 0.05 after 250 s of testing. Other publications showed slightly different values and also in comparison with these findings above. Publication [60] showed similar values of CoF with a similar configuration of the tribometer; nevertheless, different operating conditions and lubricants were tested. Publications [46,47,48] showed experiments with a similar model of synovial fluids providing similar results of CoF. Different cartilages were also tested: a cartilage removed from guinea pigs [38], bovine bones [57], and human specimens [51]. Though the samples were removed from different animals, the values and trends of CoF were comparable; nevertheless, the deviation of CoF depended on the combination of friction samples, testing conditions, etc. A rise in CoF trends, which was shown in this study, has been known from other publications [38,46,47,48,49,50,51,52,53,54,55,56,57,58,59,60], and the values of CoF were comparable depending on the processing conditions, configuration of specimens, and lubricants.

### 3.3. Methodology and its limits

All measurements were carried out with one cartilage specimen. The reason is a great variation of tribological and mechanical properties across cartilage samples removed from different bones. Mechanical properties, especially elasticity modulus, depend on the site of sample removal, which is connected with the deviation of tribological properties [72,73]. Mechanical and tribological properties are better when the specimens are removed from the most loaded site of the bone surface. Specimen properties also deviate due to the type of joint because each joint is differently loaded [17,66,74]. Tribological properties also depend on the age of the animal from which the specimen is removed [38]. The use of only one specimen declared the comparability of the percentage deviations of measurement values. The cartilage as a biological material tissue is also sensitive to degradation in air; therefore, all experiments were carried out consecutively in one day [75]. The lubricant was also a biological specimen so there was a risk of degradation. Moreover, there was also a risk of gradual loss of the fluorescence properties of the dye contained in the lubricant specimens. When the lubricant was excited by the light source, the emitted light from the dye gradually decreased. It could be assumed that the measured fluorescence intensity, which was the basis for the evaluation of the particle count by the new software, was thus affected. 

Real natural synovial joints operate under various conditions (load, sliding speed between cartilages) depending on the type of movement (walk, trot, run, etc.) [73]. The direction and type of movement between the surfaces of cartilages vary depending on the type of joint (knee, hip, etc.); each joint operates under special kinematic conditions [73,76,77]. The present study used a simplified model of the synovial joint. The kinematic operating conditions were simplified to the reciprocating motion with a constant velocity (see Figure 5); i.e., the kinematic operating conditions were simplified from the multi-axis motion with various velocity and load to the single-axis motion with constant velocity and load. This simplified model of the synovial joint with a reciprocating tribometer used the cartilage as a testing specimen and the glass plate as the friction specimen. The glass plate ensured insight into the contact; however, the modulus of elasticity of glass is of a larger order than that of cartilage tissue. The performed experiments used model synovial fluids that did not contain all of the components of physiological synovial fluid. The compositions of the fluids were adapted to the possibilities of fluorescence labelling of synovial fluid components. Previous research studies used different fluid compositions; moreover, each animal has a unique synovial fluid composition. 

## 4. Conclusions

The present research showed a new approach to cartilage friction and lubrication evaluation. Friction measurement was connected with simultaneous visualization of cartilage contact, which helped to better understand the cartilage lubrication processes. This opened a new look at the evaluation of individual components of the lubricant and the correlation with the friction coefficient. The newly designed evaluation software and experimental device were presented. The evaluation of lubrication was based on the processing of the contact record provided by this software, which evaluated records (snaps) from the fluorescence microscope. Friction measurements simultaneously with visualization were carried out for on-off loaded contact and the reciprocating test with the model synovial fluid. The conclusions from the measurements were as follows:

The on-off loaded contact showed a decreasing trend of particle count in the contact, which pointed to weeping of lubricant out of the contact. 

The albumin protein played a major role in lubrication and created a stable lubrication film in the contact.

The connection between the rising trend of friction and the trend of albumin particle count was indicated. 

The ϒ-globulin protein showed a significant decrease of particle count in the contact, which pointed to its smaller role in the cartilage lubrication. 

This methodology represented great potential for understanding the lubrication system in human synovial joints, which will help to treat joint diseases. Our research assumed the future experiments to be focused on the analysis of the impact of individual components of synovial fluid on lubrication and friction, and also the rehydration of cartilage will be examined. The newly designed experimental apparatus together with the newly developed evaluation methodology could open new possibilities for testing of other soft contacts, such as contact lenses, rubbers, or soft polymers. 

## Figures and Tables

**Table 1:** Lubricants’ composition.

Lubricant	Albumin (mg/mL)	ϒ-globulin (mg/mL)	HA (mg/mL)	Labelled Component
Model Synovial Fluid 1	20	3.6	2.5	Albumin
Model Synovial Fluid 2	20	3.6	2.5	ϒ-globulin
Calibration Fluid 1	20	-	-	Albumin

**Table 2:** Experimental conditions.

Load	Contact Pressure	Velocity	Stroke	Total Distance	Number of Cycles	Duration	Temperature
10 N	0.8 MPa	10 mm/s	20 mm	1200 mm	60	approximately 4.5 min	37 °C

**Table 3:** Percentage deviation of CoF.

Lubricant	Mask Radius (pixel)	Contact Center x/y (pixel)	Top Hat	Threshold	Labelled Component	Fluorescence Filter
Model Synovial Fluid 1	1000	1300/1100	15	4	Albumin	TRITC
Model Synovial Fluid 2	1000	1300/1100	13	4	ϒ-globulin	FITC

**Table 4:** Evaluation outputs from experiments: percentage deviations of measured magnitude.

Lubricant	Deviation of CoF	Deviation of Particle Count
Model Synovial Fluid 1	61.55%-Increase	3.73%-Decrease
Model Synovial Fluid 2	56.96%-Increase	23.33%-Decrease
